# A Two-Step Ferric Chloride and Dilute Alkaline Pretreatment for Enhancing Enzymatic Hydrolysis and Fermentable Sugar Recovery from Miscanthus sinensis

**DOI:** 10.3390/molecules25081843

**Published:** 2020-04-16

**Authors:** Lingci Li, Peng Ye, Mengyu Chen, Shangyuan Tang, Ying Luo, Yifan Gao, Qiong Yan, Xiyu Cheng

**Affiliations:** College of Life Sciences and Bioengineering, School of Science, Beijing Jiaotong University, Beijing 100044, China; 18121614@bjtu.edu.cn (L.L.); 17121611@bjtu.edu.cn (P.Y.); 18272032@bjtu.edu.cn (M.C.); 16121638@bjtu.edu.cn (S.T.); 19121606@bjtu.edu.cn (Y.L.); 19121602@bjtu.edu.cn (Y.G.)

**Keywords:** two-step pretreatment, *Miscanthus sinensis*, enzymatic hydrolysis, sugar recovery

## Abstract

A two-step process was proposed to enhance enzymatic hydrolysis of *Miscanthus sinensis* based on a comparative study of acid/alkaline pretreatments. Ferric chloride pretreatment (FP) effectively removed hemicellulose and recovered soluble sugars, but the enzymatic hydrolysis was not efficient. Dilute alkaline pretreatment (ALP) resulted in much better delignification and stronger morphological changes of the sample, making it more accessible to enzymes. While ALP obtained the highest sugar yield during enzymatic hydrolysis, the soluble sugar recovery from the pretreatment stage was still limited. Furthermore, a two-step ferric chloride and dilute alkaline pretreatment (F-ALP) has been successfully developed by effectively recovering soluble sugars in the first FP step and further removing lignin of the FP sample in the second ALP step to improve its enzymatic hydrolysis. As a result, the two-step process yielded the highest total sugar recovery (418.8 mg/g raw stalk) through the whole process.

## 1. Introduction

Energy, environmental pollution, public health and food safety are the most important issues for global sustainable development, and production of renewable energy from different organic wastes is attracting increasing attention [1,2,3,4,5,6,7,8]. Lignocellulosic biomass (e.g., corn stalk, wheat straw, and rice straw), which is a sustainable and renewable energy source with reduced net CO_2_ emission, has been widely investigated as substrates for ethanol and biogas fermentation [8,9]. In addition to these biomass wastes, energy crops are also potential candidates for biofuel production [10,11]. For instance, *Miscanthus sinensis,* which is a perennial grass, is throughout most of provinces in China. As a typical energy crop, its attractive advantages include high yield (27~38 t/ha), easy propagation, effective nutrient cycling, and high genetic variation [4,12,13]. While various agricultural wastes were widely investigated for biofuel production, studies on the energy crops (e.g., *Miscanthus sinensis*) were still limited. Considering its potential, *Miscanthus sinensis* was chosen as a model energy crop to study its application prospects.

One of the major drawbacks when lignocellulosic biomass is being converted is its recalcitrant structure. Various methods, including steam explosion, hot water, organic solvent, acid, and alkaline pretreatments, have been investigated for improving bioconversion efficiency of lignocellulosic biomass [2,8,14,15,16,17,18,19]. An ideal pretreatment process aims 1) to improve enzymatic hydrolysis by dissolving lignocellulosic components and changing microstructure, 2) to minimize the loss of sugars and the formation of by-products for an efficient sugar recovery in the pretreatment step, and 3) to reduce the energy demands and the cost [14,15,16,17,18,19]. Although a number of pretreatment techniques operated at severe conditions (e.g., steam explosion and hot water pretreatment, 160–240 °C, 5–45 min, pretreatment severity factor (log R_0_) of 2.8–4.8) were effective at enhancing enzymatic hydrolysis of the pretreated samples, they had high energy consumption and produced enzyme-inhibiting by-products as compared to those observed at mild conditions (e.g., acid pretreatment, 120 °C, 5–45 min, log R_0_ of 1.3–2.2) [4,20,21]. Some other processes with less by-product formation and energy requirement (e.g., biological pretreatment at room temperature) can also improve bioconversion, but these processes are typically time-consuming and/or consume sugars of raw stalks during the pretreatment [2,11]. Up to now, there is no one strategy that could well meet all requirements for the ideal pretreatment.

Among different techniques, acid and alkaline pretreatments were widely considered as promising candidates [22,23,24,25]. On the one hand, acid pretreatment, in which H_2_SO_4_ or HCl are commonly used, has been found to be relatively inexpensive, and to obtain high hemicellulose recoveries and/or digestibility of different wastes such as Napier grass, bamboo, and stalk wastes [24,25]. In spite of their successful application for many biomass wastes, acid pretreatments in some other cases failed to significantly enhance the enzymatic hydrolysis of the pretreated stalks [24]. Previous studies have shown that mild acid pretreatment using Lewis acids such as FeCl_3_ also enhanced the enzymatic hydrolysis of the pretreated biomass [11,26]. The corrosion problem in traditional acid pretreatments could also be weakened by using Lewis acids. On the other hand, alkaline pretreatment, which improves cellulose digestibility by providing the effective delignification and chemical swelling of fibrous cellulose, is considered as one of the current leading pretreatment methods [25]. Enzymatic hydrolysis of various biomass wastes such as bamboo, Eucalyptus samples, and pine foliage was significantly enhanced by alkaline pretreatments [17,23,25]. However, it should also be noted that the dissolved cellulosic components in the alkaline pretreatment can’t be well recovered due to the formation of by-products [27]. These results necessitated further studies to investigate the real mechanism.

In this work, different pretreatments were investigated for identifying an ideal pretreatment process and evaluating the potential of *Miscanthus sinensis* biomass in the bioconversion industry. Firstly, ferric chloride pretreatment (FP) and dilute alkaline pretreatment (ALP) were studied to enhance cellulosic substrate depolymerization and sugar recovery. The changes of chemical composition and morphological structure of the substrates were then investigated to better illustrate the corresponding mechanisms. Finally, a two-step ferric chloride and dilute alkaline pretreatment (F-ALP) was further developed for efficient recovery of total fermentable sugars through the whole process from the pretreatment to the enzymatic hydrolysis.

## 2. Materials and Methods

### 2.1. Materials

Sun-dried *Miscanthus sinensis* samples were obtained from Zhangzhou, Fujian province, China. These samples were dried in an oven at 60 °C for more than 24 h to a constant weight. A miller was then used to mill them to pass through a 20-mesh sieve. The cellulose, hemicellulose, and lignin contents of *Miscanthus sinensis* were 41.7%, 28.3%, and 20.3%, respectively.

### 2.2. Pretreatment Operation

One-step processes including FP and ALP were conducted in glass bottles by an antoclave. In brief, the dried *Miscanthus sinensis* samples were added to the glass bottles containing 0.8–4.8% (mass/volume percent concentration, *w/v*) FeCl_3_ solutions and 0.4–1.2% (*w/v*) NaOH for FP and ALP, respectively [11,22,26,28]. A solid loading rate of 10% was chosen. The above *Miscanthus sinensis* mixtures for all three pretreatments were treated in an autoclave at 121 °C for 30 min. The pretreated mixtures were centrifuged and the supernatants were collected for further analysis. The solid residues were washed with deionized water until the filtrates were neutral. The solids were then dried in an oven at 105 °C until a constant weight was observed. The dried solids were kept in a desiccator at room temperature.

In the first step of two-step ferric chloride and dilute alkaline pretreatment (F-ALP)process, the dried *Miscanthus sinensis* samples were added to the glass bottles containing 1.6% or 3.2% (*w/v*) FeCl_3_ solutions based on a solid loading rate of 10%. The above *Miscanthus sinensis* mixtures were then autoclaved at 121 °C for 30 min. The solid residues of the pretreated mixtures were washed and then added to glass bottles containing 1.0% or 1.2% (*w/v*) NaOH based on a solid loading rate of 10% for the second step pretreatment. The above mixtures were then autoclaved at 120 °C for 30 min. The solid residues and supernatants after the second step pretreatment were then collected and stored for further analysis and/or the subsequent enzymatic hydrolysis based on the same procedure in the one-step processes.

### 2.3. Enzymatic Hydrolysis of Stalk Samples

A 250-mL conical flask using 50-mM sodium acetate buffer (pH 5) containing 40 μL tetracycline hydrochloride (25 mg/mL) was used for the enzymatic hydrolysis of the pretreated samples [9]. The stalk samples were added on the basis of a 2.5% solid loading and the enzymatic hydrolysis was then performed at 50 °C and a 150-rpm agitation rate for 72 h. The cellulase obtained from Hunan Youtell Biochemical Co., Ltd. (Hunan province, China) was used for the enzymatic hydrolysis. The enzyme loading ratio was 15 FPU/g of stalk substrate (FPU, filter paper unit). One FPU is defined as the amount of the enzyme that produces glucose from filter paper substrates at 1 μmol/min in the reaction mixture at 50 °C and pH 5. Reducing sugars in the enzymatic hydrolysate were determined by standard method of the 3, 5-dinitrosalicylic acid (DNS) assay [29].

The “cellulase amount used (CAU)” (FPU/g) represents the amount of cellulase used through the whole pretreatment-enzymatic hydrolysis process of one-gram raw stalks, and it was calculated with the following equation:

CAU (FPU/g) = L*Y(1)

where CAU is the cellulase amount used, L is the enzyme loading ratio (15 FPU/g), and Y is the solid yield of each pretreatment (%).

### 2.4. Analysis

The sample mixtures were centrifuged after different pretreatments. The supernatants were collected to analyze total solid, solid yield, and total soluble sugar content according to the standard methods [30,31,32]. Total soluble sugar content was determined by a phenol–sulfuric acid method [31,32]. Solid yields were calculated on the basis of residual total solid of stalk samples after all pretreatments. The cellulose, hemicellulose, and lignin contents were determined gravimetrically according to the standard method of Goering and Van-Soest and calculated on the basis of residual total solid [11,33]. In brief, the neutral detergent fiber (NDF) content was determined gravimetrically by extracting the solid residue of different pretreatments with neutral detergent (ND). The acid detergent fiber (ADF) content was then determined gravimetrically by extracting the solid residue of the ND extraction with acid detergent (AD). Lignin content was then determined gravimetrically from the free ash after the solid residue of AD extraction was extracted using sulfuric acid solution (72%). Cellulose content was obtained by subtracting the pre-ash lignin level from the ADF level. The ADF level was subtracted from the NDF level to obtain hemicellulose content. Ash content of the solid residue was measured gravimetrically in a muffle furnace at 550 °C over 6 h [11,33]. All experiments were done in triplicate.

Pretreatment severity factor (log R_0_) was calculated on the basis of the following equation [34]:

R_0_ = t*exp[(T_r −_ 100)/14.75] (2)

where T_r_ is 100, and t are the pretreatment temperature (°C), the reference temperature (°C), and the reaction time (min), respectively. The fitted value (14.75) is the arbitrary constant w.

The microstructure of raw and the treated stalk samples of *Miscanthus sinensis* with 3.2% FeCl_3_ or 1.0% NaOH was observed by scanning electron microscopy (SEM) [28]. A specimen holder with aluminum tape was used to fix the dried stalk samples. Gold was then sputtered to the surface of the samples using a JEOL JEC-1200 sputter-coater (Tokyo, Japan). These specimens were then examined with a JEOL JSM-5600 LV scanning electron microscope (Tokyo, Japan) under high vacuum. An accelerating voltage of 5.0 kV was used for the observations. 

## 3. Results and Discussion

### 3.1. Effect of Ferric Chloride Pretreatment (FP) and Dilute Alkaline Pretreatment (ALP) on Composition

FP and ALP were used to pretreat the *Miscanthus sinensis* samples and decrease the recalcitrance of the biomass. As shown in Table 1, the solid yields become lower in FP with higher FeCl_3_ concentrations. The weight loss could be related to the hemicellulose removal of the stalk samples [28]. The corresponding hemicellulose contents of the FP samples were obviously lower than that observed in raw stalks, indicating that the removal of hemicellulose was much easier than the removal of cellulose during pretreatment [11,22]. The cellulose content of the FP sample pretreated with 3.2% FeCl_3_ reached 58.3%. No significant increase was observed in the cellulose content of the FP sample as the pretreatment condition became severer (4.8% FeCl_3_), while the hemicellulose content further reached 9.5%. These results suggested that the removal of recalcitrant cellulose components was slightly improved in this condition. An obvious decrease of cellulose recovery from the pretreated sugarcane bagasse sample was also observed as the FeCl_3_ concentrations were higher than 0.1 mol/L, but the cellulose removal was still significantly lower as compared to hemicellulose [26]. Different acid pretreatments randomly open glycosidic bonds, dissolve hemicellulose, and improve the cellulose content [35]. Therefore, the accessibility of lignocellulosic biomass to microorganisms and cellulases is improved [35].

In the case of ALP, the solid yields were about 55.0–79.8%. Both lignin and hemicellulose were significantly removed. As the NaOH concentration was 1.2%, the hemicellulose content reached 14.2%. The corresponding cellulose content was as high as 62.5% in ALP with 1.2% NaOH. As compared to FP, ALP with 1.0–1.2% NaOH indicated more significant delignification effect and the lignin contents decreased to 12.0–13.9% (Table 1). Effective delignification was also observed in different alkaline pretreatments of many other stalk wastes [19,23,27].

Dissolved cellulosic components will be partly converted into soluble sugars. As shown in Table 1, the highest soluble sugar level in the aqueous phase of the pretreated mixtures by ALP reached 93.7 mg/g raw stalk (RS). It should be noted that the level of soluble sugars in the case of FP was much higher than that observed in ALP (256.9 mg/g RS vs. 93.7 mg/g RS, i.e., 163 mg/g RS more in FP), while the solid yields were slightly lower (52.9% vs. 55.0%, i.e., 529 mg/g RS vs. 550 mg/g RS, 21 mg/g RS less in FP). The higher soluble sugar yield in FP could not be mainly due to the lower solid yield but less by-product formation such as formic acid and acetic acid as compared to ALP [27]. The acid pretreatment can markedly convert hemicellulose into monomeric sugars and soluble oligomers, and reduced sugar loss can be achieved with mild acid at mild operational conditions [11,28].

### 3.2. Effect of FP and ALP on Enzymatic Hydrolysis

**Figure 1:**
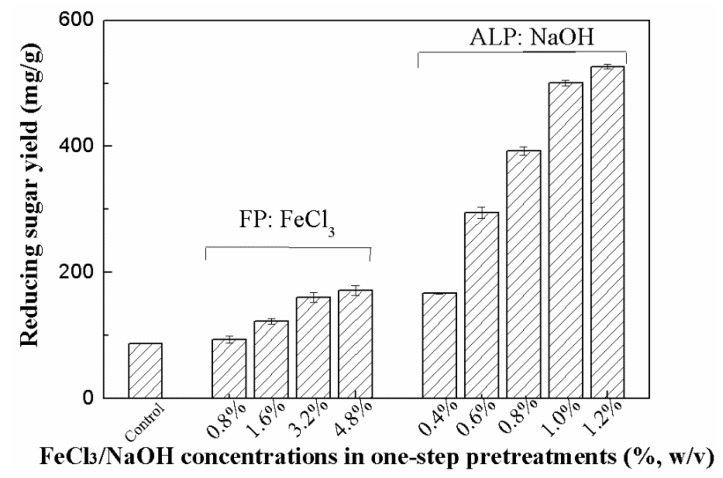
Enzymatic hydrolysis of the stalk samples.

Enzymatic hydrolysis of the untreated sample, FP, and ALP samples was determined and the results are shown in Figure 1. The untreated *Miscanthus sinensis* sample indicated the lowest reducing sugar yield (86.9 mg/g RS) after 72 h of the enzymatic hydrolysis because of its recalcitrant structure. After FP and ALP, the enzymatic hydrolysis of the pretreated samples was enhanced. In the case of FP, the reducing sugar yield of the stalk sample pretreated with 0.8% FeCl_3_ solution was 93.8 mg/g pretreated stalk (PS). As the FeCl_3_ concentrations were higher, the sugar yields become higher (93.8 mg/g PS vs. 160.7 mg/g PS, *p* < 0.05). No significant enhancement was observed in the reducing sugar yield of the FP sample as the pretreatment condition became severer (4.8% FeCl_3_). 

ALP appeared to be a more efficient pretreatment technique for improving the enzymatic hydrolysis of the pretreated samples. Results in Figure 1 indicate that the reducing sugar yield of the stalk sample pretreated with very dilute NaOH solution (0.6% NaOH) was 294.6 mg/g PS, which is obviously higher than those observed in all FP samples. The highest sugar yield in ALP reached as high as 526.5 mg/g PS, which is 506% higher than the untreated sample. 

Enzymatic hydrolysis of lignocellulosic biomass wastes was significantly impeded by its recalcitrance, including the dense structure of cell wall, lignin barrier, and coating effect of hemicellulose [17]. In this work, a positive relationship was observed between the hemicellulose removal and the reducing sugar yields in the enzymatic hydrolysis of the FP samples (Table 1 and Figure 1). It could be due to the fact that removing the hemicellulose components increases the cellulose accessibility [19]. FP were effective at removing the hemicellulose components, but the reducing sugar recovery in the enzymatic hydrolysis of the FP samples was still not very high. Limited improvement of the hydrolysis efficiency was also observed in previous studies [24,36]. Reducing sugar yield of bamboo pretreated by dilute acid was only 77.0 mg/g stalk (9.5% of hydrolysis yield), while very low glucose yields of 7–11% from dilute acid pretreated bamboo fractions were reported in another study [24,36].

Interestingly, the delignification also exhibited a positive relationship with the reducing sugar yields of the ALP samples in the enzymatic hydrolysis stage (Table 1 and Figure 1). Although hemicellulose removal was relatively lower, ALP showed more significant delignification (lignin content of the pretreated samples: 12% vs. 21.4–23.5%) and much higher reducing sugar recovery as compared to FP (526.5 mg/g PS vs. 171.0 mg/g PS). The present study agreed well with previous findings in which enzymatic hydrolysis of different lignocellulosic wastes such as bamboo, energy crop samples, Eucalyptus samples, and pine foliage were significantly improved after efficient removal of lignin by alkaline pretreatments [11,16,23,25]. Some ester bonds and glycosidic linkages can be degraded or broken by alkaline pretreatments. These changes will cause the decrease of lignin–hemicellulose complex, the lignin removal, cellulose swelling, etc., thereby producing the considerable enhancement of digestibility [16,17,23,24]. Together with the above observations, the present results suggest that the lignin removal, which helps decrease unproductive binding of enzymes to lignin and improve the cellulose accessibility [19], appears to be one of the most important factors, causing the higher sugar release in ALP.

### 3.3. Effect of FP and ALP on Structure

**Figure 2:**
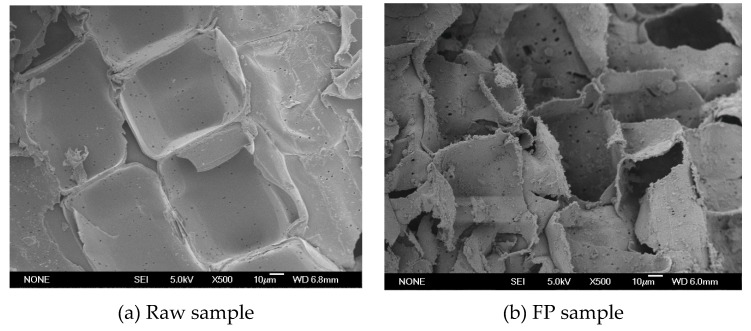

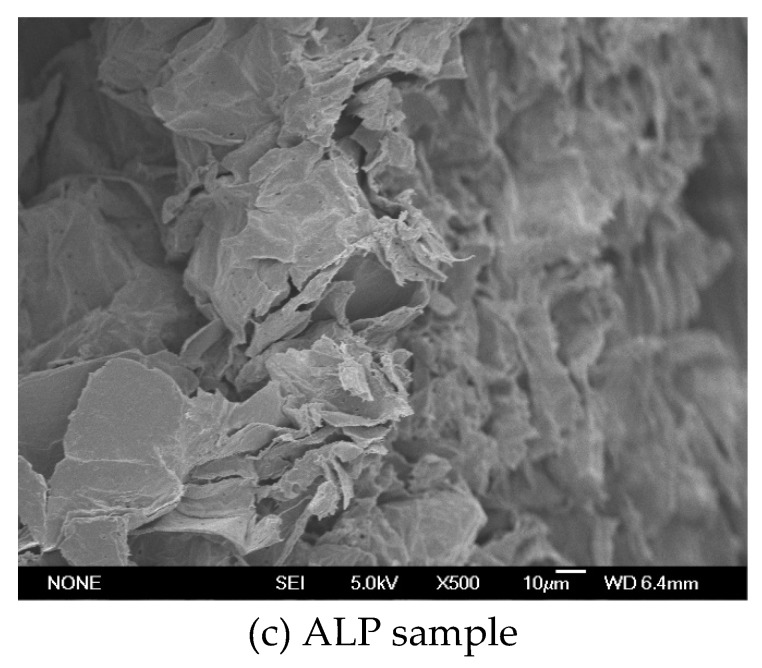
SEM observations of the *Miscanthus sinensis* samples before and after pretreatments (500×). (**a**) Raw sample; (**b**) ferric chloride pretreatment (FP) sample; (**c**) alkaline pretreatment (ALP) sample.

Morphological structure changes of the untreated and pretreated samples were observed using a scanning electron microscope at a magnification ratio of 500×. As shown in Figure 2a, the untreated stalk sample had a rigid surface structure, which could impede the accessibility of cellulose to cellulases. By contrast, distinct differences were observed in the FP and ALP samples as compared to the untreated sample (Figure 2b,c). As shown in Figure 2b, the cell surfaces of the FP sample were not smooth and exhibited some cracks and small particle-sized debris. Some big porous structures appeared on the surface of the FP sample (Figure 2b). These results indicated that the removal of cellulosic components such as hemicellulose by FP produced important morphological modification. Enhanced destruction of microstructure was also found in some lignocellulosic wastes with acid pretreatments [11,28]. There is a correlation between morphological changes and the operational conditions because of the difference of diverse mechanisms on removal of lignocellulosic components. Both FP and ALP significantly removed hemicellulose components and destroyed the original dense structure of the biomass, hence improving enzyme action on the corresponding samples (Figure 1, Figure 2, and Table 1). 

As compared to FP, the microstructure changes of the ALP sample were quite significant. Figure 2c shows that the surface of the ALP sample was remarkably destroyed so that the rectangular cell wall boundaries became extremely distorted and blurry. It was possible that the strong delignification effect and efficient removal of hemicellulose of the stalk sample were obtained by ALP. Meanwhile, separated fibers, particle-sized debris, and distorted cell wall structures emerged in the ALP sample. Delignification, which decreased the adsorption of cellulase onto lignin, also resulted in stalk substrates with higher cellulose content [16,17,23]. All these changes released large amounts of reactive sites on the surface of cell walls in the ALP sample. As a result, the ALP sample become more accessible to enzymes in the subsequent hydrolysis and the high reducing sugar yield was achieved.

### 3.4. Effect of the Two-Step Process on Sugar Recovery

Notably, the sugar recovery from the aqueous phase of the pretreated mixtures by FP reached the highest level of 256.9 mg/g RS. However, the subsequent enzymatic hydrolysis efficiency of the FP sample was not very attractive (Table 1 and Figure 1). By contrast, the enhancement of enzymatic hydrolysis efficiency by ALP was remarkable, in spite of the fact that the sugar recovery from the dissolved cellulosic components in the pretreatment stage was not very high (93.7 mg/g RS). Therefore, a two-step process coupling FP with a post-treatment of ALP was proposed to further improve the total fermentable sugar recovery. 

**Figure 3:**
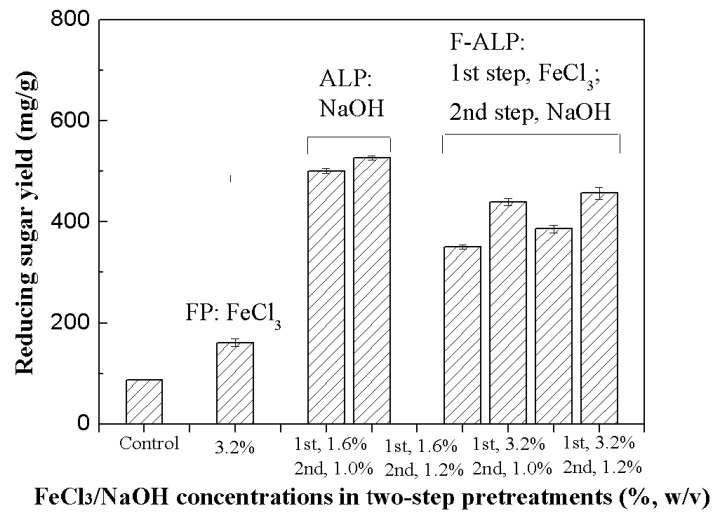
Enzymatic hydrolysis of the ferric chloride and dilute alkaline pretreatment (F-ALP) samples.

In the two-step ferric chloride and dilute alkaline pretreatment (F-ALP), easily degradable hemicellulose components were effectively recovered from the first FP step as we did in the one-step FP process (Table 1). The solid residue from the first FP step was then subjected to the second ALP step to remove lignin, which is expected to enhance the subsequent enzymatic hydrolysis. In this study, the FP samples obtained from two conditions (1.6% and 3.2% FeCl_3_, 120 °C, 30 min) were further treated in the second ALP step with 1.0% or 1.2% NaOH, respectively. The F-ALP samples were then collected for the following enzymatic hydrolysis. Results in Figure 3 indicate that the reducing sugar level in the enzymatic hydrolysate of the F-ALP sample from relatively mild conditions (step 1, 1.6% FeCl_3_; step 2, 1.0% NaOH) reached as high as 350.7 mg/g PS, increasing by 118% as compared to that obtained in the one-step FP process. Further enhancement was observed when the amounts of FeCl_3_ or NaOH addition in FP and ALP increased. The highest reducing sugar yield of 457.0 mg/g PS was achieved in the enzymatic hydrolysis of the F-ALP sample.

### 3.5. Mass Balance for One-Step/Two-Step Pretreatments and Enzymatic Hydrolysis Processes

An ideal pretreatment process not only enhances digestibility of the pretreated sample, but also maximizes the recovery of soluble sugars in the whole process. The mass balance of the stalk samples under different pretreatments and the subsequent enzymatic hydrolysis was calculated. The results are shown in Table 2. The highest soluble sugar yield of the pretreatment stage was obtained in FP (4.8% FeCl_3_, 256.9 mg/g RS), against 93.7 mg/g RS observed in ALP. However, the corresponding soluble sugar recovery from the enzymatic hydrolysis of the FP sample was 171.0 mg/g PS, which only accounted for 26% of the total fermentable sugar recovery of 347.4 mg/g RS. By contrast, the fermentable sugar recovery in the enzymatic hydrolysis of the ALP sample (ALP with 1.2% NaOH) reached as high as 526.5 mg/g PS, which accounted for 76% of the total fermentable sugar recovery (383.3 mg/g RS). The highest total fermentable sugar recovery of 390.3 mg/g RS was obtained by ALP with relatively lower alkali addition (1.0% NaOH) due to the higher solid yield in the pretreatment stage as compared to ALP with 1.2% NaOH.

In the case of the two-step process, the first FP step indicated a good balance of dissolving cellulosic components and reducing soluble sugar loss in the pretreatment stage, and the second ALP step successfully enabled to obtain the readily degradable substrates. As a result, the soluble sugar yields from the FP step, the ALP step, and the enzymatic hydrolysis of the F-ALP sample were 240.2 mg/g RS, 32.9 mg/g PS, and 457.0 mg/g PS, respectively. The corresponding total soluble sugar yield reached the highest level of 418.8 mg/g RS, increasing by another 104% as compared to that achieved in the one-step FP process. It is also higher than the highest sugar recovery achieved in one-step ALP processes. Moreover, another striking observation to emerge from the data comparison was that much more fermentable sugars were recovered from the pretreatment stage of the two-step process rather than the enzymatic hydrolysis stage as compared to one-step ALP (62% vs. 23%), thereby resulted in a substantial decrease of cellulase amount used (about 36%). It is well known that the bottleneck of bioethanol industry is the high costs, in which cellulase cost (about 0.50 USD/gallon) accounts for 20–30% of total costs [37]. Increasing attention has been attracted to decreasing the cost of the bioenergy industry by optimizing enzyme production, pretreatment methods, saccharification, and fermentation processes [38,39,40,41,42]. The enzymatic hydrolysates of the pretreated stalks and the pretreatment hydrolysates (mainly dissolved hemicellulosic components) can be used for ethanol and xylitol fermentation by *Saccharomyces cerevisiae* and *Candida tropicalis*, respectively [43]. Economics of ethanol fermentation from lignocellulosic biomass could be supported by co-production of high-value xylitol, which is an important product in food and pharmaceutical industries [43].

The total fermentable sugar recovery from the *Miscanthus sinensis* samples pretreated by the two-step F-ALP was comparable to those obtained in the enzymatic hydrolysis of varied lignocellulosic substrates as well as *Miscanthus* species [4,15,23,25]. Maximum released sugar yields of 146.9–588 mg/g PS was obtained from various biomass wastes (e.g., wild rice grass, *Pennisetum* grass, and bamboo) pretreated by acid and alkaline pretreatments [11,15,23]. Total fermentable sugar yields of 370–700 mg/g PS (about 220–420 mg/g RS) have been obtained from *Miscanthus sinensis* biomass samples pretreated with 4% H_2_SO_4_ or 4% NaOH at 121 °C for 20 min [44]. In this study, the total fermentative sugar yield obtained so far was as high as 418.8 mg/g RS of *Miscanthus sinensis* in the two-step F-ALP process and the corresponding conversion ratio reached 53.4%, with mild operational conditions (120 °C for 30 min, pretreatment severity factor (log R_0_) of 2.1), hence providing a potential alternative for efficient biofuel production from *Miscanthus sinensis*.

## 4. Conclusions

ALP indicated a high soluble sugar yield during the enzymatic hydrolysis, while FP was proven as an efficient method to recover soluble sugars at the pretreatment stage. Furthermore, an efficient two-step process of coupling ferric chloride with dilute alkaline pretreatment has been successfully developed by effectively recovering soluble sugars in the first FP step and further removing lignin from the FP sample in the second ALP step to improve the enzymatic hydrolysis of the F-ALP sample. As a result, the two-step F-ALP process yielded the highest total sugar recovery (418.8 mg/g raw stalk) throughout the whole pretreatment and enzymatic hydrolysis process, with a substantial reduction in the cellulase amount used. 

## Figures and Tables

**Table 1:** Solid yield and chemical composition after different pretreatments.

Pretreatments	Chemical Concentrations (%)	Solid Yield (%)	SS of PTS (mg/g RS)^1^	HC (%)	CC (%)	LC (%)
Control	--	--	--	28.3 ± 0.7	41.7 ± 0.4	20.3 ± 0.7
FP	0.8% FeCl_3_	71.5 ± 1.4	130.9 ± 11.5	13.6 ± 1.8	53.9 ± 0.9	18.7 ± 1.1
	1.6% FeCl_3_	62.8 ± 3.2	174.8 ± 5.9	11.3 ± 1.5	55.3 ± 0.8	19.7 ± 1.3
	3.2% FeCl_3_	54.9 ± 1.6	240.2 ± 9.3	10.6 ± 1.4	58.3 ± 0.7	20.5 ± 1.7
	4.8% FeCl_3_	52.9 ± 2.1	256.9 ± 8.5	9.4 ± 1.7	58.8 ± 1.1	21.4 ± 1.6
ALP	0.4% NaOH	79.8 ± 1.0	52.7 ± 0.7	26.6 ± 0.9	49.6 ± 0.8	18.9 ± 0.6
	0.6% NaOH	75.6 ± 1.0	56.0 ± 1.1	20.5 ± 1.2	51.4 ± 1.4	16.5 ± 0.5
	0.8% NaOH	62.0 ± 0.9	70.4 ± 1.0	15.4 ± 0.7	57.6 ± 0.6	15.6 ± 0.4
	1.0% NaOH	59.8 ± 1.3	90.7 ± 1.4	14.6 ± 0.6	59.1 ± 0.3	13.8 ± 0.1
	1.2% NaOH	55.0 ± 0.6	93.7 ± 0.5	14.2 ± 0.6	62.5 ± 0.5	12.0 ± 0.3

Note: ^1^ Soluble sugar (SS) is the level of soluble sugar in the liquid of the pretreatment mixtures. HC: hemicellulose content; CC: cellulose content; LC: lignin content; SS: soluble sugars; RS: raw stalk; PTS: pretreatment stage.

**Table 2:** Mass balance of the stalk samples.

Single/Two-Step Pretreatments			SS Yield of PTS		SS Yield of EH	Total SS Yield	Cellulase Amount Used^2^
	Step 1	Step 2	Step 1 (mg/g RS)	Step 2 (mg/g PS)	(mg/g PS)	(mg/g RS)^1^	(FPU/g)
Control	--	--	--	--	--	86.9	15
FP	3.2% FeCl_3_	--	240.2	--	160.7	328.4	8.2
	4.8% FeCl_3_	--	256.9	--	171.0	347.4	7.9
ALP	0.8%NaOH	--	70.4	--	392.4	313.7	9.3
	1.0%NaOH	--	90.7	--	501.0	390.3	9.0
	1.2%NaOH	--	93.7	--	526.5	383.3	8.3
F-ALP	1.6% FeCl_3_	1.0%NaOH	174.8	31.2	350.7	353.0	6.8
	1.6% FeCl_3_	1.2%NaOH	174.8	32.6	439.7	376.1	6.2
	3.2% FeCl_3_	1.0%NaOH	240.2	32.0	386.4	408.8	5.9
	3.2% FeCl_3_	1.2%NaOH	240.2	32.9	457.0	418.8	5.3

Note: ^1^The total soluble sugar yield through the whole process was calculated on the basis of raw stalk. SS: soluble sugars; RS: raw stalk; PS: pretreated stalk; PTS: pretreatment stage; EH: enzymatic hydrolysis. ^2^ The “cellulase amount used” (FPU/g) represents the amount of cellulase used through the whole pretreatment–enzymatic hydrolysis process of one-gram raw stalks (it was calculated with Equation (1) in Section 2.3).

## References

[1] Duque A., Manzanares P., González A., Ballesteros M. (2018). Study of the application of alkaline extrusion to the pretreatment of Eucalyptus biomass as first step in a bioethanol production process. Energies.

[2] Amiri H., Karimi K. (2018). Pretreatment and hydrolysis of lignocellulosic wastes for butanol production: Challenges and perspectives. Bioresour. Technol..

[3] Xie L., Zhao S.S., Rogers K.M., Xia Y.A., Zhang B., Suo R., Zhao Y. (2020). A case of milk traceability in small-scale districts-Inner Mongolia of China by nutritional and geographical parameters. Food Chem..

[4] Fu S.F., Chen K.Q., Zhu R., Sun W.X., Zou H., Guo R.B. (2018). Improved anaerobic digestion performance of Miscanthus floridulus by different pretreatment methods and preliminary economic analysis. Energ. Convers. Manage..

[5] Zhao Y., Zhang B., Guo B., Wang D.H., Yang S.M. (2016). Combination of multi-element and stable isotope analysis improved the traceability of chicken from four provinces of China. CYTA-J. Food.

[6] Li J.B., Zhang H.Y., Lu M.S., Han L.J. (2019). Comparison and intrinsic correlation analysis based on composition, microstructure and enzymatichydrolysis of corn stover after different types of pretreatments. Bioresour. Technol..

[7] Zhao Y., Zhang B., Chen G., Chen A.L., Yang S.M., Ye Z.H. (2014). Recent developments in application of stable isotope analysis on agro-product authenticity and traceability. Food Chem..

[8] Kucharska K., Rybarczyk P., Holowacz I., Lukajtis R., Glinka M., Kamiński M. (2018). Pretreatment of lignocellulosic materials as substrates for fermentation processes. Molecules.

[9] He Y.C., Zhang D.P., Di J.H., Wu Y.Q., Tao Z.C., Liu F., Zhang Z.J., Chong G.G., Ding Y., Ma C.L. (2016). Effective pretreatment of sugarcane bagasse with combination pretreatment and its hydrolyzates as reaction media for the biosynthesis of ethyl (S)-4-chloro-3-hydroxybutanoate by whole cells of E-coli CCZU-K14. Bioresour. Technol..

[10] Phitsuwan P., Sakka K., Ratanakhanokchai K. (2016). Structural changes and enzymatic response of Napier grass (Pennisetum purpureum) stem induced by alkaline pretreatment. Bioresour. Technol..

[11] Tang S.Y., Xu C.M., Khanh Vu L.T., Liu S.C., Ye P., Li L.C., Wu Y.X., Chen M.Y., Xiao Y., Wu Y. (2019). Enhanced enzymatic hydrolysis of Pennisetum alopecuroides by dilute acid, alkaline and ferric chloride pretreatments. Molecules.

[12] Brandt A., Gräsvik J., Hallett J.P., Welton T. (2013). Deconstruction of lignocellulosic biomass with ionic liquids. Green Chem..

[13] Kang K.E., Park D.H., Jeong G.T. (2013). Effects of inorganic salts on pretreatment of Miscanthus straw. Bioresour. Technol..

[14] Jin S., Zhang G., Zhang P., Li F., Wang S., Fan S., Zhou S. (2016). Microwave assisted alkaline pretreatment to enhance enzymatic saccharification of catalpa sawdust. Bioresour. Technol..

[15] Sahoo D., Ummalyma S.B., Okram A.K., Pandey A., Sankar M., Sukumaran R.K. (2018). Effect of dilute acid pretreatment of wild rice grass (Zizania latifolia) from Loktak Lake for enzymatic hydrolysis. Bioresour. Technol..

[16] Wang W.H., Zhang C.Y., Tong S.S., Cui Z.Y., Liu P. (2018). Enhanced enzymatic hydrolysis and structural features of corn stover by NaOH and ozone combined pretreatment. Molecules.

[17] Wang Z.N., Hou X.F., Sun J., Li M., Chen Z.Y., Gao Z.Z. (2018). Comparison of ultrasound-assisted ionic liquid and alkaline pretreatment of Eucalyptus for enhancing enzymatic saccharification. Bioresour. Technol..

[18] Xia F., Gong J.W., Lu J., Cheng Y., Zhai S.R., An Q.D., Wang H.S. (2019). Combined liquid hot water with sodium carbonate-oxygen pretreatment to improve enzymatic saccharification of reed. Bioresour. Technol..

[19] Meng X.Z., Wells T., Sun Q.N., Huang F., Ragauskas A. (2015). Insights into the effect of dilute acid, hot water or alkaline pretreatment on the cellulose accessible surface area and the overall porosity of *Populus*. Green Chem..

[20] Kim D. (2018). Physico-chemical conversion of lignocellulose: Inhibitor effects and detoxification strategies: A mini review. Molecules.

[21] Michelin M., Ximenes E., de Lourdes Teixeira de Moraes Polizeli M., Ladisch M.R. (2016). Effect of phenolic compounds from pretreated sugarcane bagasse on cellulolytic and hemicellulolytic activities. Bioresour. Technol..

[22] Camesasca L., Ramı’rez M.B., Guigou M., Ferrari M.D., Lareo C. (2015). Evaluation of dilute acid and alkaline pretreatments, enzymatic hydrolysis and fermentation of napiergrass for fuel ethanol production. Biomass Bioenergy.

[23] Li H., Chen X., Wang C., Sun S., Sun R. (2016). Evaluation of the two-step treatment with ionic liquids and alkali for enhancing enzymatic hydrolysis of Eucalyptus: Chemical and anatomical changes. Biotechnol. Biofuels.

[24] Li K.N., Wan J.M., Wang X., Wang J.F., Zhang J.H. (2016). Comparison of dilute acid and alkali pretreatments in production of fermentable sugars from bamboo: Effect of Tween 80. Ind. Crops Products.

[25] Pandey A.K., Negi S. (2015). Impact of surfactant assisted acid and alkali pretreatment on lignocellulosic structure of pine foliage and optimization of its saccharification parameters using response surface methodology. Bioresour. Technol..

[26] Zhang H.D., Lyu G.J., Zhang A.P., Li X., Xie J. (2018). Effects of ferric chloride pretreatment and surfactants on the sugar production from sugarcane bagasse. Bioresour. Technol..

[27] Dziekonska-Kubczak U., Berłowska J., Dziugan P., Patelski P., Pielech-Przybylska K., Balcerek M. (2018). Nitric Acid pretreatment of jerusalem artichoke stalks for enzymatic saccharification and bioethanol production. Energies.

[28] Liu C.Z., Cheng X.Y. (2010). Improved hydrogen production via thermophilic fermentation of corn stover by microwave-assisted acid pretreatment. Int. J. Hydrogen Energy.

[29] Miller G.L. (1959). Use of dinitrosalicylic acid reagent for determination of reducing sugar. Anal. Chem..

[30] APHA (1998). Standard Methods for the Examination of Water and Wastewater.

[31] SEPAC (2002). The Methods for Water and Wastewater Monitoring and Analysis.

[32] Dubois M., Gilles K.A., Hamilton J.K., Rebers P.A., Smith F. (1956). Colorimetric method for determination of sugars and related substances. Anal Chem..

[33] Goering H.K., Van-Soest P.J. (1970). Agricultural Handbook No. 379. Forage Fiber Analyses, Apparatus, Reagents, Procedures and Some Applications.

[34] Pedersen M., Meyer A.S. (2010). Lignocellulose pretreatment severity-relating pH to biomatrix opening. New Biotechnol..

[35] Brienzo M., Fikizolo S., Benjamin Y., Tyhoda L., Görgens J. (2017). Influence of pretreatment severity on structural changes, lignin content and enzymatic hydrolysis of sugarcane bagasse samples. Renew. Energy.

[36] Xin D., Yang Z., Liu F., Xu X., Zhang J. (2015). Comparison of aqueous ammonia and dilute acid pretreatment of bamboo fractions: Structure properties and enzymatic hydrolysis. Bioresour. Technol..

[37] Du J. (2010). Novozymes accelerates cellulosic ethanol commercialized. China WTO Tribune.

[38] Puligundla P., Smogrovicova D., Mok C., Obulam V.S.R. (2018). A review of recent advances in high gravity ethanol fermentation. Renew. Energy.

[39] Zhang B., Sun Q., Liu H.J., Li S.Z., Jiang Z.Q. (2017). Characterization of actinidin from Chinese kiwifruit cultivars and its applications in meat tenderization and production of angiotensin I-converting enzyme (ACE) inhibitory peptides. LWT-Food Sci. Technol..

[40] Sun Q., Zhang B., Yan Q.J., Jiang Z.Q. (2016). Comparative analysis on the distribution of protease activities among fruits and vegetable resources. Food Chem..

[41] Nosrati-Ghods N., Harrison S.T.L., Isafiade A.J., Tai S.L. (2018). Ethanol from biomass hydrolysates by efficient fermentation of glucose and xylose-A Review. Chembioeng. Rev..

[42] Zhang B., Liu Y., Yang H.Y., Yan Q.J., Yang S.Q., Jiang Z.Q., Li S.Z. (2017). Biochemical properties and application of a novel beta-1,3-1,4-glucanase from *Paenibacillus barengoltzii*. Food Chem..

[43] Si S.L., Chen Y., Fan C.F., Hu H.Z., Li Y., Huang J.F., Liao H.F., Hao B., Li Q., Peng L.C. (2015). Lignin extraction distinctively enhances biomass enzymatic saccharification in hemicelluloses-rich Miscanthus species under various alkali and acid pretreatments. Bioresour. Technol..

[44] Unrean P., Ketsub N. (2018). Integrated lignocellulosic bioprocess for co-production of ethanol and xylitol from sugarcane bagasse. Ind. Crop. Prod..

